# Antiviral Effects of Artemisinin and Its Derivatives against SARS-CoV-2 Main Protease: Computational Evidences and Interactions with ACE2 Allelic Variants

**DOI:** 10.3390/ph15020129

**Published:** 2022-01-22

**Authors:** Riadh Badraoui, Mongi Saoudi, Walid S. Hamadou, Salem Elkahoui, Arif J. Siddiqui, Jahoor M. Alam, Arshad Jamal, Mohd Adnan, Abdel M. E. Suliemen, Mousa M. Alreshidi, Dharmendra K. Yadav, Houcine Naïli, Hmed Ben-Nasr

**Affiliations:** 1 Department of Biology, University of Ha’il, Ha’il 81451, Saudi Arabia; wa.hamadou@uoh.edu.sa (W.S.H.); s.elkahoui@uoh.edu.sa (S.E.); arifjamal13@gmail.com (A.J.S.); j.alam@uoh.edu.sa (J.M.A.); arshadjamalus@yahoo.com (A.J.); mo.adnan@uoh.edu.sa (M.A.); abuelhadi@hotmail.com (A.M.E.S.); mousa.algladi@gmail.com (M.M.A.); 2 Laboratory of Histology-Cytology, Medicine Faculty of Tunis, University of Tunis El Manar, La Rabta, Tunis 1007, Tunisia; 3 Laboratory of Histo-Embryology & Cytogenetics, Medicine Faculty of Sfax, University of Sfax, Sfax 3029, Tunisia; 4 Department of Life Science, Faculty of Sciences of Sfax, University of Sfax, Sfax 3000, Tunisia; mongifss@yahoo.fr; 5 Molecular Diagnostic and Personalized Therapeutics Unit, University of Ha’il, Ha’il 81451, Saudi Arabi; 6 College of Pharmacy, Gachon University of Medicine and Science, Hambakmoeiro 191, Yeonsu-gu, Incheon 21924, Korea; dharmendra@gachon.ac.kr; 7 Laboratory of Solid State, Faculty of Sciences of Sfax, University of Sfax, Sfax 3000, Tunisia; houcine_naili@yahoo.com; 8 Laboratory of Pharmacology, Medicine Faculty of Sfax, University of Sfax, Sfax 3029, Tunisia

**Keywords:** artemisinin, SARS-CoV-2, COVID-19, ACE2 allelic variants, computational approach, main protease (Mpro), molecular dynamics

## Abstract

Fighting against the emergent coronavirus disease (COVID-19) remains a big challenge at the front of the world communities. Recent research has outlined the potential of various medicinal herbs to counteract the infection. This study aimed to evaluate the interaction of artemisinin, a sesquiterpene lactone extracted from the *Artemisia* genus, and its derivatives with the SARS-CoV-2 main protease. To assess their potential use against COVID-19, the interactions of the main active principle of *Artemisia* with the severe acute respiratory syndrome coronavirus 2 (SARS-CoV-2) main protease (Mpro) was investigated through in silico probing. Our results showed that artemesinin and its derivatives manifested good oral absorption and bioavailability scores (0.55). They potently bound to the Mpro site of action—specifically, to its Cys145 residue. The selected compounds established two to three conventional hydrogen bonds with binding affinities ranging between −5.2 and −8.1 kcal/mol. Furthermore, artemisinin interactions with angiotensin converting enzyme 2 (ACE2) were dependent on the ACE2 allelic variants. The best score was recorded with rs961360700. A molecular dynamic simulation showed sufficient stability of the artemisinin–Mpro complex on the trajectory of 100 ns simulation frame. These binding interactions, together with drug-likeness and pharmacokinetic findings, confirmed that artemisinin might inhibit Mpro activity and explain the ethnopharmacological use of the herb and its possible antiviral activity against SARS-CoV-2 infection inducing COVID-19. Nevertheless, it interacted differently with the various ACE2 allelic variants reported to bind with the SARS-CoV-2 spike protein.

## 1. Introduction

The coronavirus infectious disease (COVID-19), due to the emergent severe acute respiratory syndrome coronavirus 2 (SARS-CoV-2) infection, has caused over 2.236 million deaths in the world. Although great efforts have been spent to fight COVID-19, finding specific and efficacious treatments is still a central challenge for medical and scientific communities [1,2], and different strategies have been envisaged to combat the disease [3]. As urgently required, newly developed anti-SARS-CoV-2 vaccines are being delivered with unprecedented clinical phase cascade overtum. While showing first-to-second-phase effectiveness in controlling the disease, their secondary outcomes are still debated, specifically within the emergent RNA vaccine type [4,5]. Within the rapid mutability and tremendous appearance of new variants of the SARS-CoV-2 virus, vaccines might lose their specificities and performance [6,7]. In our opinion, classical treatments, like the antimalarial hydroxychloroquine that has been efficaciously used by several infectious centers and has been approved in treating the disease by many authors [8,9,10], are sought as ideal for relieving the infection and saving lives. Alternative medicine also showed great potential in fighting COVID-19 [11,12].

COVID-19 results from a SARS-coronavirus infection that has a single-stranded genome encoding for two specific functional enzymes, a papain-like protease (PL-pro) and a 3C-like protease (3CL-pro) that is also called Main protease (Mpro). Both enzymes are translated by the host-cell ribosomes. These two enzymes govern a proteolytic process producing 16 nonstructural proteins in need for the virus replication. PL-pro cuts the viral RNA-expressed polyprotein into three sites and the later accomplishes the cleaving process at 11 different sites [13]. The Main virus protease (Mpro) is a 306 amino acid chain with a 3-chymotrypsin-like protease activity that comprises three domains. Its substrate binding site is settled in a pocket region formed by two domains and presents a catalytic dyad (Cys145/His41) [14,15]. Its activation is processed by transferring a proton from Cys145 to His41, an acylation–diacylation intermediate state and interactions with peptide substrates [16]. Due to its importance in viral replication, Mpro is sought as an ideal target for developing new drugs inhibiting coronaviruses—in particular, the endemic emergent COVID-19 [17,18,19]. Recent data show proof of the antiviral effects of various herbal medicines that constitute a potential chemical scaffold to inhibit this enzyme [20,21,22,23,24]. Other investigations focused on targeting the papain-like protease of SARS-CoV-2 [25,26,27]. Among these medicinal plants, *Artemisia* shrubs (Asteraceae family), which were assigned as safe in several clinical trials [28,29,30,31], were considered with great interest for fighting COVID-19 [32]. These herbs have been used by several populations to cure influenza, cough, and respiratory distress [33]. In an experimental model of lung microbial infection, Yang and colleagues (2015) [34] showed that the essential oil of *Artemisia* efficiently inhibits microbial replication and biofilm formation that perturbs the pulmonary architecture; it was suggested that such effects might be a consequence of both their antiviral and antimicrobial activities. Molecular screening revealed the presence of several chemicals in the herb extracts that exhibit virucidal activity, such as artemisinin, rutin, and kaempferol. In particular, artemisinin, a sesquiterpene lactone, and its derivatives were shown to efficiently treat malaria. They also exert substantial antiviral activity [35] and were also shown to bind the SARS-CoV-2 spike protein at Lys353, thereby inhibiting the spike protein binding to angiotensin-converting enzyme 2 (ACE2) receptors of the host cells [36]. Nevertheless, their efficiency remains dependent on ACE2 receptor polymorphism. Several studies reported that SARS-CoV-2 mediates ACE2 receptor and serine protease TMPRSS2 for cellular entry and S protein priming, respectively [1,37,38]. Further investigations have proven that artemisinin derivatives interact with various cellular receptors, such as the VEGFR-1 [39,40], glucocorticoid [41] and probably the ACE2 receptors [42,43]. Taken together, these experimental findings suggest the potential of this medicinal plant genus to alleviate or neutralize COVID-19 through multiple pathway mechanisms.

In the current work, the inhibitory potential of five artemesinin derivatives on Mpro was investigated using in silico probing methods and comparisons to chloroquine as a reference drug. Molecular dynamics (MD) was carried for 100 ns as a validation approach for the complex artemisinin–SARS-CoV-2 Mpro. The molecular interactions of artemisinin with the ACE2 allelic variants, which have been proven to interact with coronaviruses, were also assessed. Furthermore, the ADMET properties, the drug-likeness, and the pharmacokinetic properties of these compounds were assessed to clarify the myth or the reality about *Artemisia* herbs use against COVID-19.

## 2. Results

**Figure 1:**
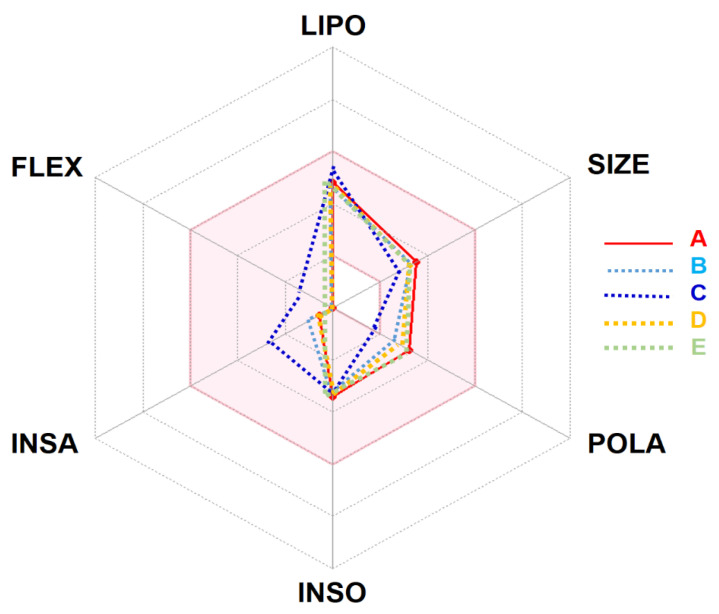
Bioavailability radar of *Artemisia campestris* selected compounds (A: artemisinin, B: acetate of dihydroartemisinin, C: artemisinic aldehyde, D: deoxyartemisinin, and E: dihydroartemisinin) based on physicochemical indices ideal for oral bioavailability. LIPO, Lipophilicity: −0.7 < XLOGP3 < þ 5; SIZE, Molecular size: 150 g/mol < mol. wt. < 500 g/mol; POLA, Polarity: 20 Å2 < TPSA <130 Å2; INSO, Insolubility: 0 < Log S (ESOL) < 6; INSA, Insaturation: 0.25 < Fraction Csp3 < 1; FLEX, Flexibility: 0 < Number of rotatable bonds < 9. The colored zone is the suitable physicochemical space for oral bioavailability. Note: the selected compounds stand in the pink area, which indicates the ideal and suitable compounds’ bioavailability.

This in silico ADMET study revealed that the selected compounds possessed the same physicochemical properties, lipophilicity, drug-likeness, and pharmacokinetics. The studied compounds manifested a good oral absorption and bioavailability score (0.55) together with an acceptable TPSA value and consensus Log Po/w. The likeness behavior and properties allow the *Artemisia* studied compounds to be orally absorbed and, thus, fall in the colored area of the bioavailability radar/polygon (Figure 1).

The table also displayed also high gastrointestinal (GI) absorption and blood–brain barrier (BBB) permeant. These selected *Artemisia* compounds were predicted to inhibit the CYP1A2 enzyme outside of the screened cytochrome P450 isoforms: CYP 1A2, CYP2C19, CYP2C9, CYP2D6, and CYP3A4. However, these compounds are not suitable for transdermal delivery (Log Kp = −5.96) (Table 1). Furthermore, Table 1 shows that the assessed compounds are not P-gp substrates, which means that there will be no problems regarding their excretion.

**Figure 2:**
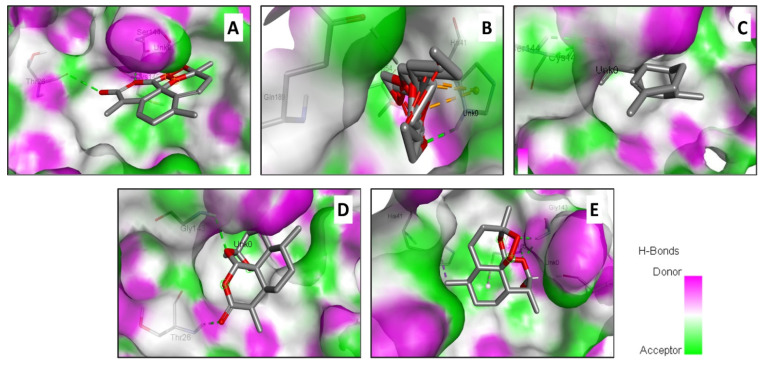
Illustration of the H-bond acceptor and donor interactions and the 3D-binding conformation of the *Artemisia campestris* selected compounds ((**A**) artemisinin, (**B**) acetate of dihydroartemisinin (**C**) artemisinic aldehyde, (**D**) deoxyartemisinin, and (**E**) dihydroartemisinin) docked to the pocket region of the SARS-CoV-2 main protease (6LU7).

In an attempt to rationalize the antiviral effect, a molecular docking assay was carried out between artemisinin derivatives encountered in *Artemisia* extract and the Mpro of SARS-CoV-2 inducing COVID-19 (Figure 2).

**Figure 3:**
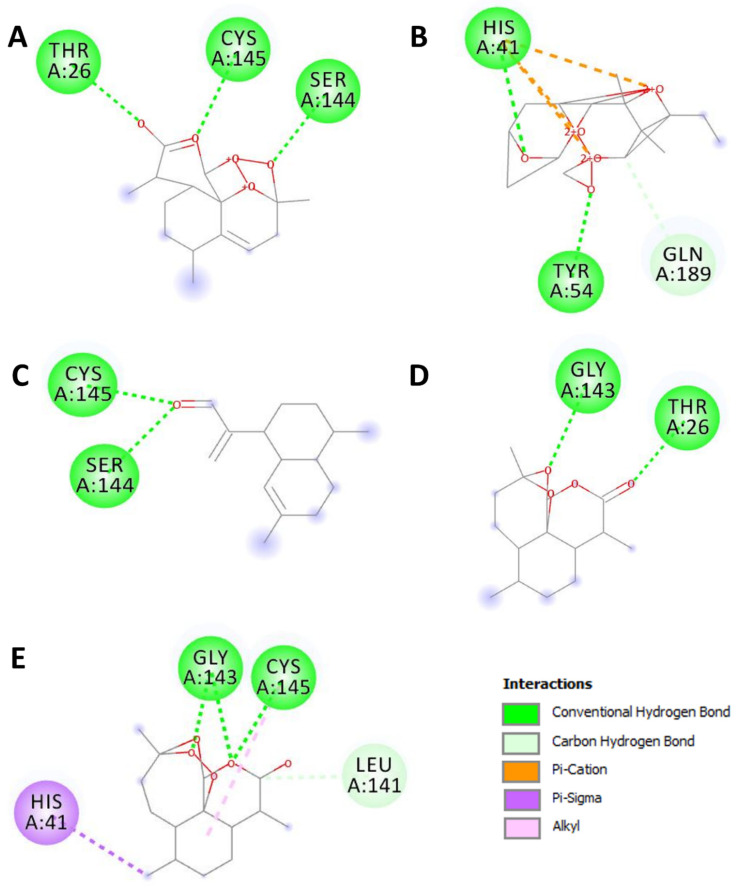
2D diagrams of the closest interactions between the *Artemisia campestris* selected compounds ((**A**) artemisinin, (**B**) acetate of dihydroartemisinin, (**C**) artemisinic aldehyde, (**D**) deoxyartemisinin, and (**E**) dihydroartemisinin) and the main protease (Mpro) of SARS-CoV-2 (6LU7) inducing COVID-19.

Table 2 exhibits the binding results. All the assessed compounds showed negative binding energy ranging from −5.2 to −8.1 kcal/mol. The highest binding score was found with the acetate of dihydroartemisinin (−8.1), followed by artemisinin (−7.2). The latter exhibited the highest number of conventional hydrogen bonds, similar to dihydroartemisinin and the reference compound. Regardless of artemisinic aldehyde, all the herb selected compound showed better affinities than the referenced drug: chloroquine (Table 2). These hydrogen bounds are represented in Figure 3.

A molecular docking analysis was also used to predict the binding affinities between artemisinin and the seventeen ACE2 variants reported to bind with the coronavirus spike protein. The binding affinities ranged between −4.9 and −8.2 kcal/mol for rs766996587 and rs961360700, respectively (Table 3).

**Figure 4:**
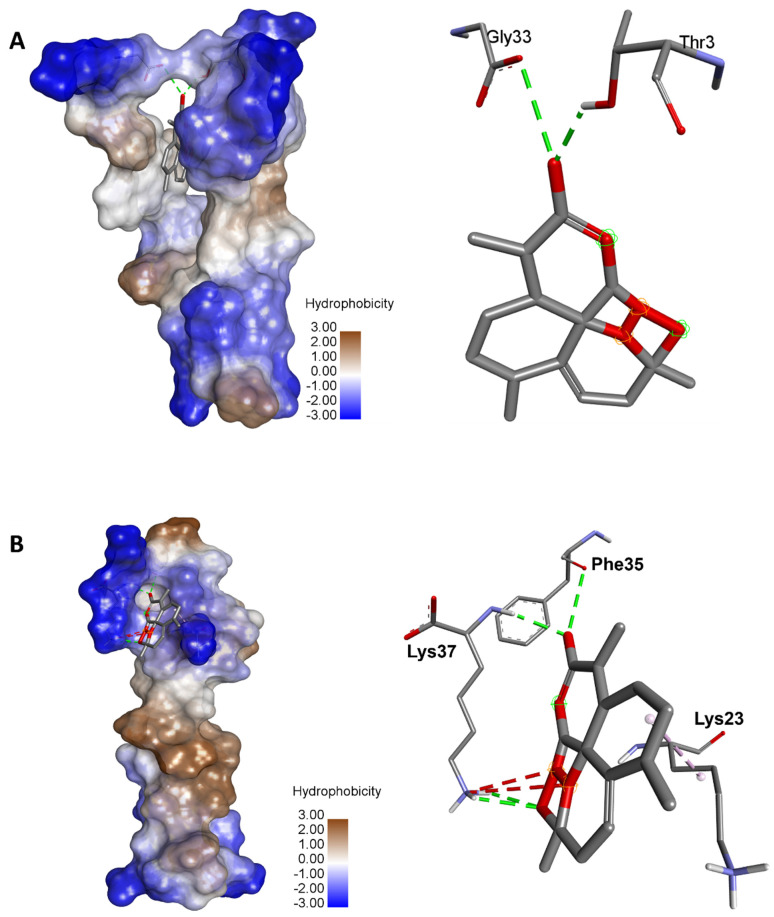
Illustration of the 3D complexes of ACE2 and artemisinin (left) and the artemisinin interactions with the amino acid residues (right). (**A**) Variant 8 of ACE2 (rs961360700) with the highest binding energy (–8.2 kcal/mol). (**B**) Variant 10 of ACE2 (rs1316056737) with the highest number of hydrogen bonds (*n* = 4).

The binding energies were supported with a network of conventional H-bond, carbon H-bond, attractive charge, alkyl, Pi-alkyl, and Pi-cation. The complex of the variant rs1316056737 artemisinin showed the highest number of conventional H-bonds (*n* = 4). Artemisinin was H-bonded to key amino acids: once to Phe35 and three times to Lys37 (Figure 4). The acetate of dihydroartemisinin, which exhibited the highest binding score with SARS-CoV-2 Mpro (−8.1 kcal/mol), also showed acceptable binding scores and interactions with the different ACE2 variants reported to bind with coronaviruses. The results are shown in Figure S1 (Supplementary File). The acetate of dihydroartemisinin was associated with negative binding energy (between −6.5 and −4.3 kcal/mol) for the seventeen targeted ACE2 variants. The best binding energy was predicted for the variant rs961360700 followed by rs755691167, with −6.5 and −5.6 kcal/mol, respectively. 

**Figure 5:**
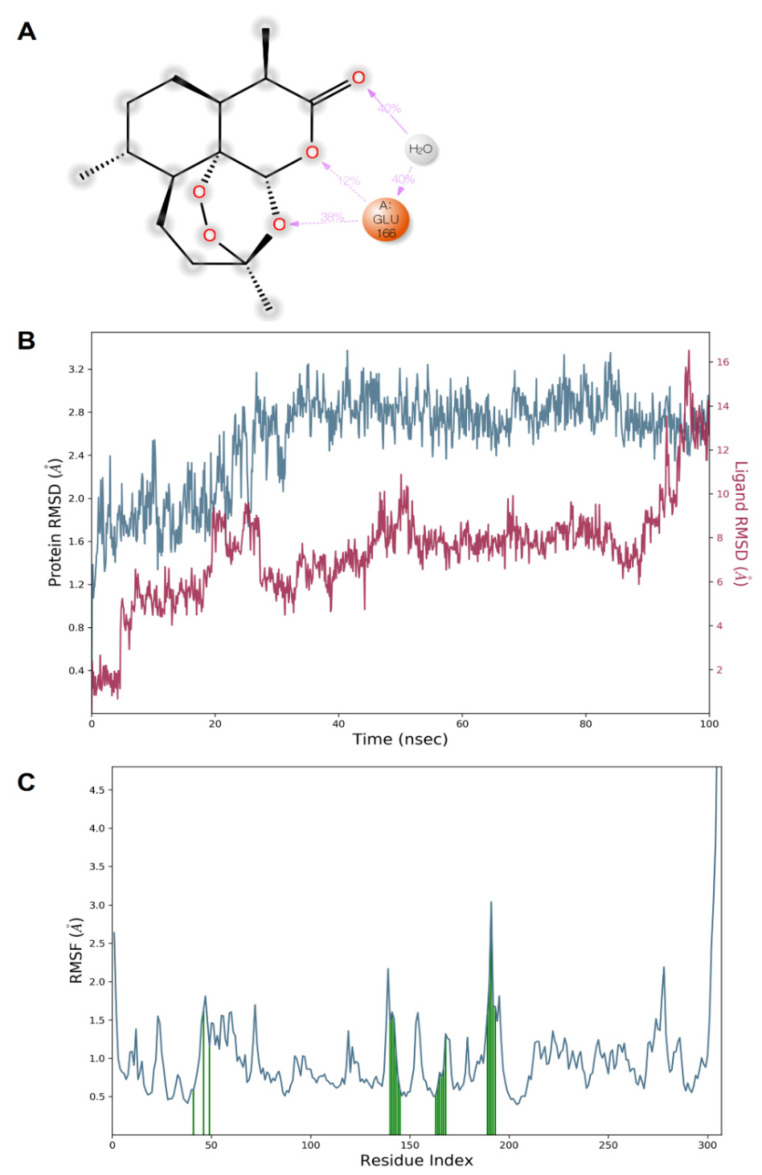
Illustration of the molecular interactions of artemisinin within the pocket region of SARS-CoV-2 Mpro (**A**). Plots of RMSD (**B**) and RMSF (**C**) values.

The dynamic simulation results for both artemisinin bound to the target (6LU7) showed that the complex was stable. The complex structure did not fall apart and remained bound throughout the simulations. The protein–ligand complex stability was further evaluated by measuring the root mean square deviation (RMSD) and root mean square fluctuations (RMSFs) to determine the fluctuation/thermal motion in the protein residues during the simulation. The RMSD plot for 6LU7 is shown in Figure 5 and gives an overview of the protein conformational perturbation during the binding. The RMSD analysis showed a little fluctuate, probably to lower the binding affinity at the 6LU7-binding site. The RMSD plot revealed that, after 20 ns, the system attained equilibrium and then oscillated further with RMSD of 0.4 Å and 2.8 Å, respectively. The fluctuations in the 6LU7 protein residues were analyzed by backbone atom motions and local changes in the secondary structure elements. The RMSF plot (Figure 5) shows that, despite both inhibitors showing the same binding amino acid residues, larger backbone residue fluctuations were seen for a complex system of artemisinin.

**Figure 6:**
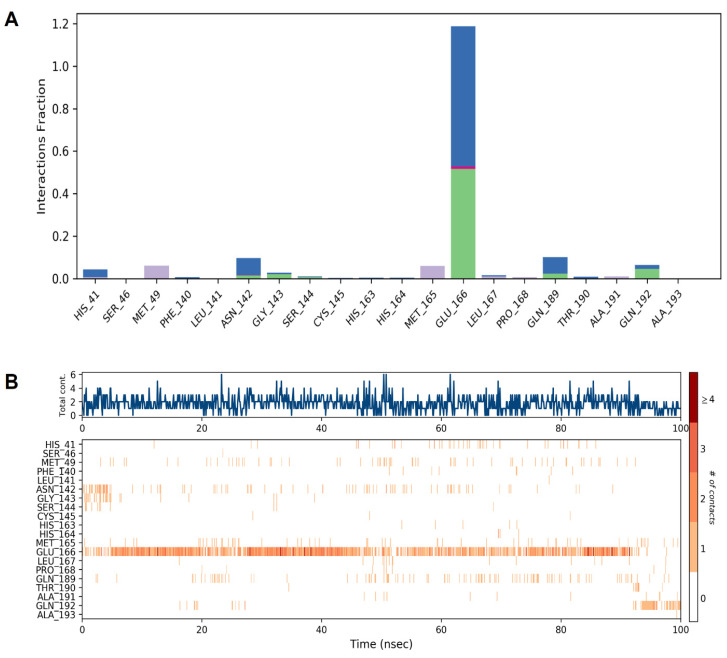
Illustration of the interaction fractions within the artemisinin and SARS-CoV-2 Mpro during a 100 ns simulation and the involved residues ((**A**) and (**B**), respectively).

The docking and MD simulation results revealed that multiple bonds (H-bond or Halogen bond) have been formed with the receptor, and its stability was further analyzed in a dynamic simulation trajectory. The dynamic simulation trajectory revealed that both the inhibitors retained their Halogen bond with a backbone atom of the Glu166 residue. The high frequency of interactions with the Glu166 residue is shown in Figure 6.

## 3. Discussion

Since the COVID-19 fight is still an ongoing challenge, an in silico study with a focus on the molecular interactions of *Artemisia* sesquiterpene lactones with the Mpro of SARS-CoV-2 was performed. The five artemisinin derivatives showed negative binding energy. Such interactions suggest that the *Artemisia* antiviral effect [34] might involve Mpro inhibition. The highest binding energies was observed when using the acetate of dihydroartemisinin that presents a binding score of −8.1 kcal/mol. However, the best docking interaction to the Mpro pocket region was found with artemisinin and dihydroartemisinin that present an additional conventional hydrogen bond. Similar to chloroquine, both artemisinin and dihydroartemisinin interacted with the Cys145 residue. Cys145 was also found to establish a conventional H-bond with artemisinic aldehyde. Similar results were reported by Omar and colleagues (2020) [43]. These interactions confirmed that the antiviral effect of artemisinin and its derivates is possible. Nobel Prize Laureate, Professor Youyou Tu, discovered artemisinin, which was extracted from the Chinese Qinghao (*Artemisia annua*), and proved its efficacy and safety to eradicate malaria [44]. More recently, this ‘magic Qinghao’ revealed further therapeutic potential, including infections by SARS coronaviruses treatment. In fact, this plants genus (*Artemisia*) contains various chemicals that might act at different levels to cure COVID-19 in a synergetic fashion. Our in silico findings argue for such a hypothesis and give proof of artemisinin derivative inhibition of the main viral protease that is essential for SARS-CoV-2 replication.

Since *Artemisia* extracts are obviously safe for use, as reported in this study and previously reported for other chemicals [45,46], we foresee that they could be administrated by both oral and inhalation routes in order to directly target the SARS-CoV-2 that particularly colonizes in the respiratory tract. The fact that the herb contains compounds that directly interact with the viral spike protein and its host cell receptors and environment (notably, inflammatory cells and thrombocytes), the inhalation pathway of the plant extracts, in conjunction with their oral administration, is envisaged as a good approach to neutralize/inhibit the SARS-CoV-2 virus. The utilization of the total herb aqueous extract might have better pharmacological properties than separated molecules. In fact, it has been previously reported that the whole plant effect is usually much better than its separated active compounds [46,47]. Furthermore, our results confirmed the beneficial effects of phytochemical compounds [47,48,49,50].

The dynamic simulation results for artemisinin bound to the target (6LU7) exhibited a considerable stability of the complex. It was reported that, after 20 ns, the system attained equilibrium and then oscillated further, with RMSD of 0.4 Å and 2.8 Å, respectively. The fluctuations in the protein residues were analyzed by backbone atom motions and local changes in the secondary structure elements.

Our results revealed that multiple bonds (H-bond or Halogen bond) were formed with the receptor, and its stability was further analyzed in a dynamic simulation trajectory. The dynamic simulation trajectory revealed that both the inhibitors retained their Halogen bond with the backbone atom of the Glu166 residue. Our docking and dynamic simulation results corroborated with the earlier similar findings reported by de Olivera et al. [51].

The drug-likeness evaluation exhibited promising properties of the selected *Artemisia* compounds. This could explain the reported antioxidant, protective, and health-promoting effects of the plant [52,53,54]. In this study, the interactions of artemisinin with the different ACE2 variants that have been reported to bind with the coronavirus were also assessed. The in silico molecular docking results showed that artemisinin interacted differently with ACE2. While the binding energy was found to be negative for all the variants, its value was mediated by ACE2 polymorphism. It ranged between −4.9 and −8.1 kcal/mol. Together with the modes of the interactions, which are reported in Figure 4, these data confirmed the potential antiviral effect of artemisinin. Our results are comparable with those previously reported on the potential anti-COVID-19 effect of artemisinin, as well as its interaction with the SARS-CoV-2 active enzyme Mpro [55,56]. In fact, it has been reported that artemisinin showed similar interaction patterns to withaferin A, curcumin, and andrographolide with SARS-CoV-2 Mpro [56]. Likewise, artemisinin–thymoquinone hybrids were also proposed by de Oliveira et al. (2021) [51] as relevant interacting compounds with the active fraction of Mpro. The phytochemical properties of these compounds were associated in silico, and the docking results ascertained the antiviral effects of *Artemisia* herbs. Nevertheless, further in vivo assays might confirm these pharmacological potencies and the potential promising effects, particularly on SARS-CoV-2 infection inducing COVID-19.

## 4. Materials and Methods

### 4.1. In Silico ADMET and Pharmacokinetic Profiles

The physicochemical and pharmacokinetics properties of the selected *Artemisia* extracted compounds were estimated based on the ADMET (absorption, distribution, metabolism, excretion, and toxicity) properties, as previously described [47,57,58].

### 4.2. Genetic Variants of Human ACE2

The genetic variants of human ACE2 were retrieved from both gnomAD and Ensemble Genome Browser. Appropriate filters were applied to determine the coding region of the ACE2 variants. The corresponding seventeen protein sequences of the different variants, which have been previously reported to bind with SARS-CoV and SARS-CoV-2 [9,59,60], were selected for further approaches. The UniProt database and PDB-BLAST were used to identify the protein structures and their functional information. The RCSB protein data bank was then used to collect the seventeen protein variants, as previously reported [9].

### 4.3. Molecular Binding and Interactions

Five sesquiterpene lactones that originate from *Artemisia* herbs (artemisinin, acetate of dihydroartemisinin, artemisinic aldehyde, deoxyartemisinin, and dihydroartemisinin) were used to assess their possible antiviral effects. The chemical structures of these compounds and chloroquine (as a reference compound) were collected from PubChem. To this end, SARS-CoV-2 was selected, particularly the crystal structure of its main protease (Mpro), using the vina package. Later on, artemisinin was also used to assess its potential interactions with the different ACE2 variants reported to bind with the coronavirus, as previously described [9,48,49,61,62].

### 4.4. Molecular Dynamics (MD) Simulation

The MD simulation was run as previously described [63]. Briefly, the Desmond program (version 5.3) with an inbuilt OPLS3 force field was used to perform molecular dynamics (MD) simulations. The selected poses of the artemisinin and SARS-CoV-2 Mpro complex issued from molecular docking were selected for the dynamic simulation. The protein–ligand complex system was immersed in a predefined water model (TIP3P) as a solvent in an orthorhombic box of size 10 Å × 10 Å × 10 Å with periodic boundary conditions. Na^+^ and Cl^−^ ions were added to neutralize the system until reaching a 0.15-M concentration. The system was relaxed, using a predefined protocol that consisted of Steepest Descent and the limited-memory Broyden-Fletcher-Goldfarb-Shanno (LBFGS) algorithms in a hybrid manner. The simulations were conducted with a constant temperature of 310.15 K using a Nosé-Hoover chain thermostat and Martyna-Tobias-Klein (MTTK) barostat methods 39 at 1 atm of pressure with the isotropic coupling type [64].

## 5. Conclusions

The in silico investigation revealed a potent binding of artemisinin and its derivatives to the SARS-CoV-2 Mpro site of action and the stability of the complex along the assessed period of 100 ns through MD simulation. The in silico molecular docking, drug-likeness, and pharmacokinetics findings provided satisfactory evidence that artemesinin might inhibit the Mpro proteolytic process, which is a must for virus replication. It is thought that, using *Artemisia* extracts—in particular, through the inhalation pathway—is promising to fight COVID-19 and may justify its ethno–pharmaceutical use against the COVID-19 pandemic.

## Figures and Tables

**Table 1:** Absorption, distribution, metabolism, excretion, and toxicity (ADMET) properties of Artemisinin and its selected derivative compounds.

Entry	Compounds				
	A	B	C	D	E
**Properties/Lipophilicity/Drug-likeness**					
Molecular weight (g × mol^−1^)	282.33	266.33	218.33	266.33	284.35
Num. heavy atoms	20	19	16	19	20
Num. arom. heavy atoms	0	0	0	0	0
Fraction Csp3	0.93	0.87	0.67	0.93	1.00
Num. rotatable bonds	0	0	2	0	0
Num. H-bond acceptors	5	4	1	4	5
Num. H-bond donors	0	0	0	0	1
Molar Refractivity	70.38	69.71	69.24	69.29	71.34
TPSA (Å²)	53.99	36.92	17.07	44.76	57.15
Consensus Log *P*_o/w_	2.49	2.72	3.44	2.58	2.25
Lipinski’s Rule.	Yes	Yes	Yes	Yes	Yes
Bioavailability Score	0.55	0.55	0.55	0.55	0.55
PAINS	0 alert	0 alert	0 alert	0 alert	0 alert
**Pharmacokinetics**					
GI absorption	High	High	High	High	High
BBB permeant	Yes	Yes	Yes	Yes	Yes
P-gp substrate	No	No	No	No	No
CYP1A2 inhibitor	Yes	Yes	No	Yes	Yes
CYP2C19 inhibitor	No	No	Yes	No	No
CYP2C9 inhibitor	No	No	Yes	No	No
CYP2D6 inhibitor	No	No	No	No	No
CYP3A4 inhibitor	No	No	No	No	No
Log Kp (cm/s)	−5.96	−6.05	−5.01	−5.90	−5.91

A: artemisinin, B: acetate of dihydroartemisinin, C: artemisinic aldehyde, D: deoxyartemisinin, and E: dihydroartemisinin.

**Table 2:** Binding affinity, conventional hydrogen bonds and interacting residues of the main *Artemisia campestris* docked compounds into the SARS-CoV-2 Mpro (6LU7).

Compound Name	Binding Affinity (kcal/mol)	Intermolecular Interactions		
		Conventional Hydrogen Bonds	Interacting Residues in the Pocket Region of SARS-CoV-2 Mpro	Closest Residue (Distance, Å)
Artemisinin	−7.2	3	**Thr26**, **Ser144**, **Cys145**	Ser144 (2.436)
Acetate of dihydroartemisinin	−8.1	2	**His41**, **Tyr54**, Gln189	Tyr54 (2.743)
Artemisinic Aldehyde	−5.2	2	**Ser144**, **Cys145**	Cys145 (2.374)
Deoxyartemisinin	−6.5	2	**Thr26**, **Gly143**	Thr26 (2.206)
Dihydroartemisinin	−6.6	3	His41, Leu141, **Gly143**, **Cys145**	Gly143 (1.980)
Chloroquine (Reference)	−5.3	3	Leu27, His41, **Leu141**, **Ser144**, **Cys145**, Met165, Glu166	(2.815)

Bold amino acids: interacting with the correspondent compound via conventional H-bonds. Underlined amino acids: same interacting residues as for the reference compound.

**Table 3:** Binding affinity, conventional hydrogen bonds, and interacting residues of the different ACE2 domains reported to bind with the coronavirus spike protein and artemisinin.

ACE2 Allelic Variant	Intermolecular Interactions	
	Interacting Residues in the Pocket Region of ACE2 Variant (Distance, Å)	Binding Affinity (kcal/mol)
rs4646116	Conventional H-Bond: Thr41 (2.219)	−6.2
	Pi Cation: Trp37 (4.044)	
	Alkyl: Arg34 (4.897)	
	Pi-Alkyl: Trp37 (4.394)	
rs73635825	Conventional H-Bond: Ser13 (2.274), Ser13 (2.874)	−5.4
	Pi-Cation: Trp16 (4.328), Trp16 (4.514), Trp16 (4.105)	
rs146676783	Conventional H-Bond: Asn53 (2.258), Ala41 (3.359), Asn53 (3.104)	−5.2
	Carbon H-Bond: Ala41 (2.892)	
rs76289235	Conventional H-Bond: Tyr26 (2.163)	−5.7
	Pi-Cation: Tyr26 (4.375)	
rs14393283	Conventional H-Bond: Trp33 (2.921), Gln30 (3.372)	−5.8
	Attractive Charge: Glu17 (3.790), Glu17 (3.195)	
	Carbon H-Bond: Trp33 (3.338)	
rs766996587	Conventional H-Bond: Asn53 (2.254), Asn53 (2.917), Asn53 (3.017)	−4.9
rs1348114695	Conventional H-Bond: Arg21 (2.428)	−5.7
	Alkyl: Ile18 (4.943)	
	Pi-Alkyl: Phe12 (4.405)	
rs961360700	Conventional H-Bond: Thr3 (2.162), Gly33 (3.377)	−8.2
rs755691167	Conventional H-Bond: Gln54 (2.346)	−6.7
	Alkyl: Val58 (3.886)	
rs1316056737	Conventional H-Bond: Lys37 (2.363), Lys37 (2.588), Lys37 (2.632), Phe35 (3.267)	−5.1
	Alkyl: Lys23 (4.193)	
rs781255386	Conventional H-Bond: Arg25 (2.494), Lys30 (2.829)	−5.1
rs1299103394	Conventional H-Bond: Ser10 (2.222), Asp13 (3.259)	−5.4
	Carbon H-Bond: Ser10 (3.414)	
	Alkyl: Cys16 (4.680), Val24 (4.002)	
rs759134032	Conventional H-Bond: Ser57 (2.127)	−5.7
	Carbon H-Bond: Leu29 (3.680)	
rs1238146879	Conventional H-Bond: Tyr5 (3.056)	−5.4
	Pi-Cation: Tyr5 (3.898)	
rs778500138	Conventional H-Bond: Leu37 (2.078), Ser38 (2.958), Asn36 (3.219)	−5.6
	Alkyl: Leu35 (4.335)	
rs1396769231	Conventional H-Bond: Ser48 (2.586), Arg49 (2.670), Leu51 (2.417)	−5.5
rs1016777825	Conventional H-Bond: His34 (2.831)	−5.4
	Alkyl: Pro21 (4.570), Val27 (4.102), Ile29 (5.295)	

## Data Availability

Data sharing not applicable.

## Supplementary Materials

The following supporting information can be downloaded at https://www.mdpi.com/article/10.3390/ph15020129/s1: Figure S1: Variation of the binding affinity of acetate of artemisinin with the 17 different variants of ACE2 proven to bind with coronaviruses (A). 3D illustration of acetate of artemisinin bound to the variant rs961360700 of ACE2, which exhibited the best free binding energy (−6.5 kcal/mol). Note the four conventional H-bonds (represented with green interrupted lines) and the involvement of three residues: Met11, Thr26 and Trp28.
