# Supplementary material for: Antiviral Effects of Artemisinin and Its Derivatives against SARS-CoV-2 Main Protease: Computational Evidences and Interactions with ACE2 Allelic Variants
Source: Pharmaceuticals (Basel). 2022 Jan 22;15(2):129. doi: 10.3390/ph15020129 (PMC8877620; doi:10.3390/ph15020129)
Supplement: Supplementary file 1 [file pharmaceuticals-15-00129-s001.zip › pharmaceuticals-1551028-supplementary.pdf]

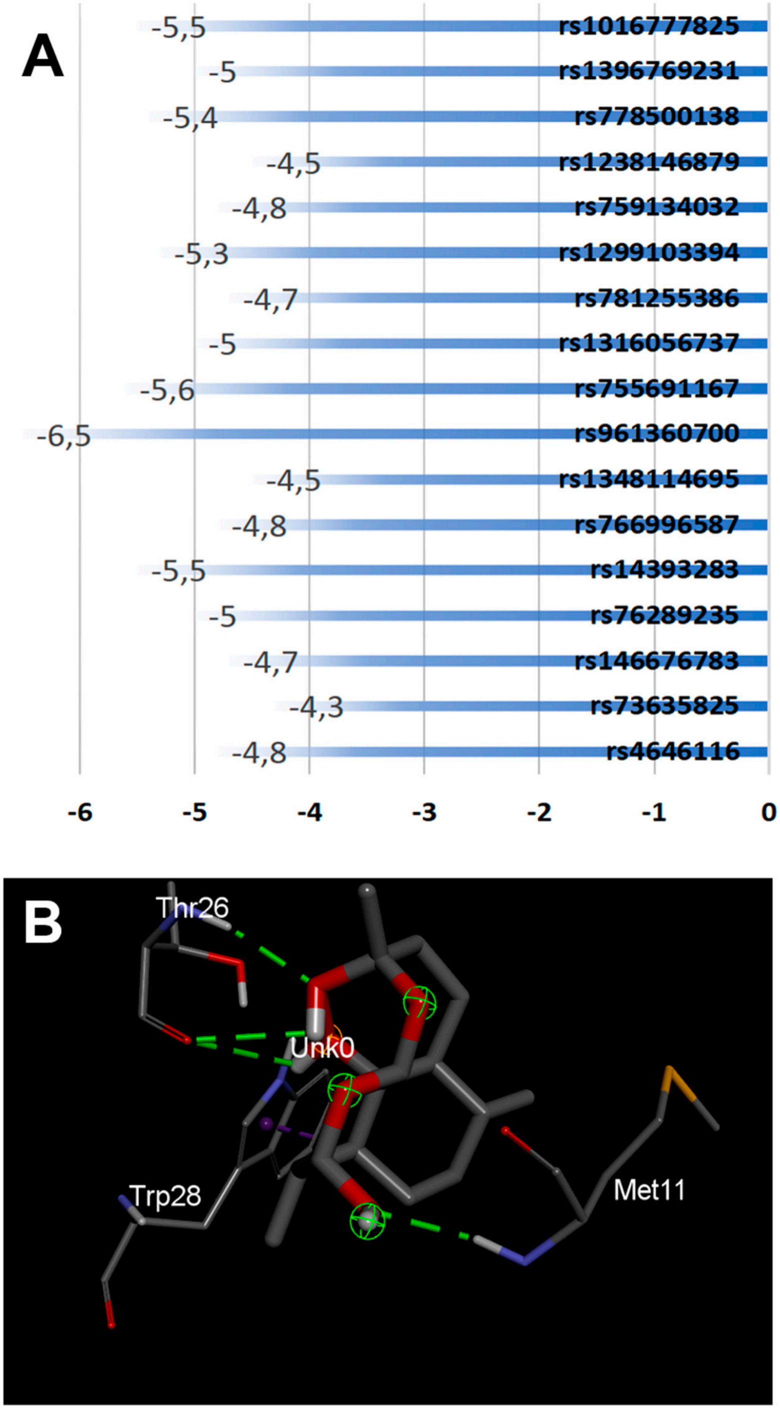

Figure S1. Variation of the binding affinity of acetate of artemisinin with the 17 different variants of ACE2 proven to bind with coronaviruses (A). 3D illustration of artemisinin bound to the variant rs961360700 of ACE2, which exhibited the best free binding energy (-6.5 kcal/mol). Note the four conventional H-bonds (represented with green interrupted lines) and the involvement of three residues: Met11, Thr26 and Trp28.
